# *Storage protein activator* controls grain protein accumulation in bread wheat in a nitrogen dependent manner

**DOI:** 10.1038/s41598-023-49139-5

**Published:** 2023-12-20

**Authors:** Anne Plessis, Catherine Ravel, Thierry Risacher, Nathalie Duchateau, Mireille Dardevet, Marielle Merlino, François Torney, Pierre Martre

**Affiliations:** 1 Université Clermont Auvergne, INRAE, UMR1095 GDEC, 63000 Clermont Ferrand, France; 2 Present Address: School of Biological and Marine Sciences, University of Plymouth, Drake Circus, Plymouth, PL4 8AA UK; 3 Centre de Recherche, Limagrain Europe, 63 720 Chappes, France; 4 Present Address: LEPSE, Université de Montpellier, INRAE, Institut SupAgro Montpellier, 34000 Montpellier, France

**Keywords:** Biochemistry, Plant sciences

## Abstract

The expression of cereal grain storage protein (GSP) genes is controlled by a complex network of transcription factors (TFs). Storage protein activator (SPA) is a major TF acting in this network but its specific function in wheat (*Triticum aestivum* L.) remains to be determined. Here we generated an RNAi line in which expression of the three *SPA* homoeologs was reduced. In this line and its null segregant we analyzed GSP accumulation and expression of GSP and regulatory TF genes under two regimes of nitrogen availability. We show that down regulation of *SPA* decreases grain protein concentration at maturity under low but not high nitrogen supply. Under low nitrogen supply, the decrease in *SPA* expression also caused a reduction in the total quantity of GSP per grain and in the ratio of GSP to albumin-globulins, without significantly affecting GSP composition. The slight reduction in GSP gene expression measured in the *SPA* RNAi line under low nitrogen supply did not entirely account for the more significant decrease in GSP accumulation, suggesting that SPA regulates additional levels of GSP synthesis. Our results demonstrate a clear role of *SPA* in the regulation of grain nitrogen metabolism when nitrogen is a limiting resource.

## Introduction

Bread wheat (*Triticum aestivum* L.) is the most important cereal crop in the world in terms of area harvested and commercial exchange. It provides on average 20% of the total protein in the human diet^1^. Wheat grain proteins have unique properties making them suitable for use in a considerable number of food and non-food products^2^. Both the total grain protein concentration (GPC) and the relative composition of the storage protein fraction govern the cohesiveness and viscoelasticity of gluten, the network formed by wheat grain storage proteins (GSPs) when mixed with water^3^. Breeding for high-yielding genotypes has decreased GPC, while modern uses of end-products require higher GPC than traditional products, limiting the potential usefulness of some varieties^4,5^. Most wheat GSPs belong to the glutenin and gliadin prolamin families. Glutenins usually account for 35–45% of total grain protein and are composed of high-molecular-weight (HMW-GS) and low-molecular-weight (LMW-GS) sub-units, which together form very large macropolymers during grain desiccation^6^. Gliadins are monomeric proteins classed as ω5-, ω1,2-, α/β- or γ-gliadins^7^ and make up between 18 and 35% of total grain protein.

Transcriptional control of GSP genes plays an important role in the endosperm specific synthesis of GSPs during cereal grain development^8^ through a network of interacting transcription factors (TFs). At least twelve TFs involved in the regulation of GSP genes have been identified in different cereal species, along with the *cis*-elements they bind to. The wheat GCN4-like motif (GLM) is bound by SPA, a basic leucine zipper TF of the Opaque2 (O2) subfamily^9,10^, with an ortholog in barley (*Hordeum vulgare* L.) named BLZ2 that binds to the same motif^11^, as does another member of the family in barley, BLZ1^12^; the ortholog of BLZ1 in wheat is SPA Heterodimerizing Protein (SHP) and is a negative regulator of GSPs^13^. Two non-homologous DNA-binding with one finger (DOF) TFs: prolamin box binding factor (PBF) and scutellum and aleurone-expressed DOF (SAD), interact with the prolamin box^14,15^; in bread wheat, PBF and SAD promote the transcription of glutenin genes by binding to the prolamin box and this activity is additive to the induction of the expression of glutenin genes by SPA^16^. In barley, an AACA motif is recognized by GAMYB (gibberellic acid-dependent of the MYB superfamily of transcriptional activators, Diaz et al. 2002). In barley, two R1MYB family TFs, Myb-related CAB promoter-binding protein (MCB1) and MYBS3, can bind a GA response complex motif^18,19^ and FUSCA3, a B3-type TF, interacts with an RY box^20^. The DOF proteins mediate the formation of several binary or ternary TF complexes^15,17,18^ in addition to the interactions between FUSCA3 and BLZ2^20^ and between BLZ1 and BLZ2^11^. The interaction of FUSCA3 with the RY box of a glutenin gene and with SPA has been demonstrated in wheat^21^. New transcription factors regulating the expression of prolamin genes have been discovered more recently in wheat: three negative regulators of GSPs, SPR and ODORANT1^22–24^, for which the exact location where they bind on prolamin promoters is still unclear, and one positive regulator, NAC109 that interacts with GAMYB^25^.

SPA/O2/BLZ2 is one of the most studied TFs in this regulatory network of grain storage proteins. In wheat, a study of different *SPA* haplotypes has shown that this gene affects the amount of nitrogen allocated to the gliadin fraction^26^. In maize, *O2* is associated with grain lysine content^27^ and an *o2* mutant shows reduced expression of some GSP genes and corresponding proteins^28^. The effect of modifying expression levels of RISBZ1, a *SPA* homolog in rice, has been tested^29,30^. Transient overexpression of *RISBZ1* in protoplasts induced trans-activation of several GSP gene promoters, which was synergistically enhanced by the simultaneous overexpression with the rice homolog of *PBF*^30^. The knockdown of *RISBZ1 in planta* caused only slight changes in GSP accumulation, but when both *RISBZ1* and *RPBF* were knocked down, GSP accumulation and gene expression were significantly reduced^29^. In wheat, the overexpression of the copy of *SPA* located on the B genome led to lower accumulation of glutenin and ω-gliadin and lower expression of *PBF*^31^.

Nutrient availability has a major effect on GSP quantity and composition^32^. Higher nitrogen input increases the amount of GSP that accumulates in the grain^33^. Differences in GSP composition related to nitrogen availability have been found to follow allometric scaling laws^34–36^, which may be a consequence of the complex transcriptional regulation network controlling GSP gene expression^26^. One particular *cis*-element on GSP gene promoters, the GLM, which is bound by SPA, plays an important role in the transcriptional response to nitrogen. It was found to be essential for the activation of GSP gene transcription in response to amino acids and ammonium, but this activation is only optimal in synergistic interaction with the endosperm box (EB), the motif formed by the GLM and the prolamin box in tandem^37^, and other *cis*-elements^38^. Interestingly, when nitrogen supply is low, the GLM may act as a negative regulatory motif for GSP gene transcription.

SPA orthologs thus have a prominent role in controlling GSP synthesis in cereals, particularly in response to changes in nitrogen availability. To confirm that SPA is a positive regulator of GSP gene expression in bread wheat and determine its role in the response of GSP accumulation and composition to nitrogen availability, plants with reduced expression of *SPA* were grown with high or low amounts of nitrogen. We analyzed the relative accumulation of the different GSP families and sub-groups and measured the expression of GSP and their regulatory TF genes throughout grain development. We show that a decrease in the expression of *SPA* caused a reduction in GPC, which was more significant under low nitrogen availability. GSP accounted for most of the decrease in GPC compared to other protein classes, with reductions in the amounts of all GSP families. We also describe the changes in regulatory TF expression that contribute to the response to nitrogen with notably *PBF, MYBS3* and *SHP* being upregulated by high nitrogen. Our results suggest that transcriptional regulation is not the sole mechanism determining the response of GSP synthesis to nitrogen availability.

## Methods

All methods were performed in accordance with the relevant guidelines and regulations.

### Plant material and growth conditions

**Figure 1:**
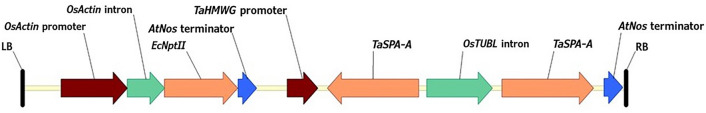
*Storage protein activator* RNA interference construct used for wheat transformation. LB, left border; RB, Right border.

Immature seeds of the spring wheat (*Triticum aestivum* L.) line NB1 (a non-commercial Spring wheat variety obtained from Limagrain Europe, Saint-Beauzire, France) were transformed by *in planta* inoculation using *Agrobacterium tumefaciens* and transgenic lines were regenerated^39^. The *SPA-A* full length coding sequence was previously amplified by PCR from a cDNA library from immature seeds of the bread wheat cultivar Récital. The binary vector pSCV was used to produce transgenic plants expressing both sense and antisense *SPA-A* cDNA separated by the first intron of rice tubulin to generate a hairpin RNA (Fig. 1). The transgene was under the control of the promoter of the subunit Dx5 of the *Glu-D1-1* HMW-GS gene and the *Nos* terminator. The plasmid includes a kanamycin resistance cassette for selection, *NptII*, controlled by the actin promoter and *Nos* terminator. For each transformation event, the number of T-DNA insertions was evaluated by qPCR performed on genomic DNA and the integrity of the transgene was verified by PCR. Transformants with several copies of the transgene were discarded. T0 plants were self-pollinated to generate the T1 generation composed of 25% homozygotes, 50% hemizygotes and 25% null segregant plants. The segregation ratio was established by cultivating 30 plants on a medium with kanamycin allowing the identification of homozygotes, hemizygotes and null segregants (Table S1). The zygosity of progenies from self-pollinated homozygotes and respective null segregants was verified by quantitative real-time PCR. Selfing of confirmed homozygotes and null segregant T2 plants gave rise to the T3 generation, i.e. the RNAi line and its null segregant to be used as a control.

T4 seeds were germinated for two to three days at room temperature on wet filter paper in Petri dishes. Germinated seeds were then transferred to soil in 50-mL PVC columns (inner diameter 7.5 cm, length 50 cm, 2 plants per column) and arranged in a greenhouse in a strip-plot design with the genotypes as rows and the nitrogen treatments (see below) as columns with four replicated blocks to form a homogeneous stand with a plant density of 261 plants m^−2^. Temperature was controlled at 22 °C during the day and 18 °C during the night. Day length was 16 h, maintained with artificial light when needed. Plants received 68 mL column^−1^ day^−1^ of water or nutrient solution. Three nutrient solutions were used to feed the plants, N0, N3 and N15. N0 contained 1 mM KH2PO4, 0.5 mM NH4NO3, 2 mM MgSO4, 7 mM CaCl2, 5 mM KCl, 10 µM H3BO3, 0.7 µM ZnCl2, 0.4 µM CuCl2, 4.5 µM MnCl2, 0.22 µM MoO3, and 50 µM EDFS-Fe; N3 contained 1 mM KH2PO4, 1 mM Ca(NO3)2, 0.5 mM NH4NO3, 2 mM MgSO4, 3 mM CaCl2, 5 mM KCl, 10 µM H3BO3, 0.7 µM ZnCl2, 0.4 µM CuCl2, 4.5 µM MnCl2, 0.22 µM MoO3, and 50 µM EDFS-Fe; N15 contained 1 mM KH2PO4, 5 mM KNO3, 4 mM Ca(NO3)2, 1 mM NH4NO3, 2 mM MgSO4, 10 µM H3BO3, 0.7 µM ZnCl2, 0.4 µM CuCl2, 4.5 µM MnCl2, 0.22 µM MoO3, and 50 µM EDFS-Fe. All plants received N3 for four weeks, then N15 until anthesis. At anthesis, continuous water irrigation was used to remove any excess of the nutrient solution in the soil; afterwards irrigation was maintained to its previous level with water. Differences in treatment between N− and N+ started at 300 °C days after anthesis, when the columns were rinsed again and lasted until grain ripeness: N+ plants received the N15 nutrient solution while N− plants received N0. Main stems were tagged when the anthers of the central florets appeared. Degree-days were calculated as the sum of the average daily temperatures after anthesis with a base temperature of 0 °C.

### Determination of grain dry mass and protein concentration

Grains from four ears (except at 200 °C days after anthesis, where five ears were used) were sampled from each replicate every 100 °C days from 200 °C days after anthesis to maturity (grain ripeness, 900 °C days after anthesis) and again at 1050 °C days after anthesis. Four grains per ear were sampled between 200 and 700 °C days after anthesis for RNA analysis and were immediately frozen in liquid nitrogen and stored at − 80 °C. The remaining grains of the ear were also frozen and stored at − 80 °C until they were freeze-dried. We measured the dry mass and nitrogen concentration of a sub-sample of grains (ca. 65%). The remaining grains were oven-dried at 80 °C for 48 h to calculate the percentage of remaining water. At maturity, grain yield per ear was calculated using all grains of each ear harvested.

Grains were milled for 2 min using a custom ball mill. Flour (5 mg) was weighed in tin capsules and the total N concentration was determined with the Dumas combustion method (Association of Analytical Communities International approved method no. 992.23) using a FlashEA 1112 N/Protein Analyzer (Thermo Electron Corp, Waltham, MA). Grain protein concentration (GPC) was calculated by multiplying grain N concentration by 5.62^40^.

### Sequential extraction, separation and quantification of grain protein fractions

Non-prolamin, gliadin and glutenin protein fractions were sequentially extracted from 60 mg of freeze-dried wholemeal flour as described by Triboi et al.^36^ and modified by Plessis et al.^41^. Each 2 mL tube contained one stainless steel bead (5 mm diameter) and samples were stirred by placing the tubes on a rotating wheel (40 rpm) during each extraction and washing step. The non-prolamin protein fraction was extracted for 30 min at 4 °C from 100 mg wholemeal flour with 1.5 mL of 50 mM phosphate buffer (pH 7.8) containing 0.1 M NaCl. After centrifugation for 10 min (18,000 g) at 4 °C, the supernatant was collected and the pellet was washed twice for 10 min each time with 1.5 mL of the same buffer. After centrifugation in the same conditions, all supernatants were pooled. The same steps were used to extract the gliadin protein fraction from the previous pellet with 70% (v/v) ethanol. Finally, the glutenin protein fraction was extracted in 50 mM borate buffer (pH 8.5) containing 2% SDS (w/v) and 1% dithiothreitol (w/v). The supernatants (80 μl) of each protein fraction were oven dried overnight at 60 °C in tin capsules and their total nitrogen concentration was determined with the Dumas combustion method as described above. Protein fractions from samples of the same flour from cultivar Récital were extracted, analysed as a control in each of the 21 sets of extractions and used to determine the coefficient of variation for each of the protein fractions, which were 3.48, 5.10, 2.19, 2.61, and 1.96% for the non-prolamin, gliadin, and glutenin protein fractions, storage proteins, and total proteins, respectively.

Gliadin classes (ω1,2-, α/β-, and γ-gliadins) and glutenin sub-units (HMW-GS and LMW-GS) were separated and quantified by HPLC (Figs. S1 and S2) using an Agilent 1290 Infinity LC system (Agilent Technologies, Santa Clara, CA, http://www.agilent.com) as described in Triboi et al.^34^. The total nitrogen concentration of each protein fraction was determined by the Dumas combustion method, as described above. The gliadin extracts used were those obtained by sequential extraction, but glutenins were extracted from flour independently with a protocol adapted from Fu and Kovacs^42^. The gliadin and glutenin extracts were filtered through regenerated cellulose syringe filters (0.45–µm pore diameter, UptiDisc; Interchim, http://www.interchim.com), and 4 µl (gliadin) or 2 µl (glutenin) of protein extract was injected into a C8 reversed-phase Zorbax 300 StableBound column (2.1 9 100 mm, 3.5 µm, 300 Å; Agilent Technologies) maintained at 50 °C. The eluents used were ultra-pure water (solvent A) and acetonitrile (solvent B), each containing 0.1% trifluoroacetic acid. The flow rate was 1 mL min^−1^. Proteins were separated by using a linear gradient, from 24 to 50% solvent B over 13 min for gliadin, and from 23 to 42% solvent B over 25 min for glutenin. Proteins were detected by UV absorbance at 214 nm. After the gradient, the column was washed with 80% solvent B for 2 min and then equilibrated at 24% (for gliadins) or 23% (for glutenins) solvent B for 2 min at the same flow rate. Chromatograms were processed with CHEMSTATION 10.1 software (Agilent Technologies). The signal obtained from a blank injection was subtracted from the chromatograms before integrating the data. The HPLC peaks corresponding to each of the four gliadin classes were identified following the observations of Wieser et al.^43^. The quantity of each gliadin class or glutenin subunit as a percentage of total gliadin or total glutenin, respectively, was calculated by dividing the areas under each HPLC peak by the total area under the chromatogram trace. The quantity of each gliadin class (or glutenin subunit) per grain was calculated by multiplying the proportion of each gliadin class (or glutenin subunit) in total gliadin (or total glutenin) by the total quantity of gliadin (or glutenin) per grain, as quantified by Dumas analysis. By subtracting the quantity of all GSPs from *N*_tot_, we calculated the amount of the remaining protein fraction, mainly constituted of albumin-globulins.

### RNA extraction and measurement of gene expression

Four grains per ear (same ears as for protein analysis) were sampled at 200, 300, 400, 500, 600 and 700 °C days, the embryos were cut out and the rest of the grain immediately frozen in liquid nitrogen and kept at − 80 °C. RNA was extracted from 75 mg of grain powder in 750 µL of extraction buffer (200 mM Tris–HCl pH 9, 400 mM KCl, 200 mM sucrose, 35 mM MgCl_2_, 25 mM EDTA) and 600 µL phenol/chloroform (pH 8). The suspension was homogenized by vortexing for 30 s and then centrifuged for 10 min at 15,000 × *g*. The supernatant was collected. The pellet was resuspended in 600 µL of phenol/chloroform, centrifuged using the same conditions and the supernatant collected, and the whole step repeated. Supernatants were pooled. RNA was precipitated by adding 1 M acetic acid (1/10 volume) and ethanol (2.5 volumes). The RNA pellet was washed with 3 M Na acetate (pH 6) and resuspended in water. A second acetic acid/ethanol precipitation was performed before resuspending the pellet in 50 µL RNase free water. RNA was treated with RNase-free DNase according to the instructions of the supplier (AMBION). The RNA in solution was quantified by measuring the absorbance at 260 nm in a spectrophotometer. Approximately 2 µg of total RNA were reverse transcribed using oligo(dT)20 and reverse transcriptase (Bio-rad iScriptTM Select cDNA Synthesis kit) in a final volume of 40 µL. Transcript levels of four housekeeping genes and the storage protein and transcription factor (TFs) genes were quantified by real-time q-PCR using Lightcycler 480 SYBR Green I Master (Roche) in 15 µL with 5 µL of cDNA diluted 10 times. Relative expression (RE) was calculated as: RE = ε^ΔCp^, where ε is the efficiency of the primers for the measured gene and ΔCp is the normalized crossing point (Cp); ΔCp = (Cp_1_ × Cp_2_ × Cp_3_ × Cp_4_)^0.25^−Cp_g_, where Cp_g_ is the Cp for the measured gene and Cp_1_, Cp_2_, Cp_3_ and Cp_4_ are the Cp values of the four housekeeping genes^44^. The primer sequences are given in Table S2.

### Soluble protein extraction and western blot analysis

Wheat flour (50 mg) from grains collected at 500 °C days after anthesis was dissolved in extraction buffer (10 mM sodium phosphate, 10 mM NaCl, pH 7.8 at 4 °C) supplemented with a protease inhibitor cocktail (P9599, Sigma, St. Louis, MO, USA). Proteins were precipitated from the extract supernatant with ice-cold acetone overnight at − 20 °C. The dried protein pellet was dissolved in SDS-PAGE buffer containing 80 mM Tris–HCl pH 8, 2% (w/w) SDS, 40% glycerol (v/v), 0.002% bromophenol blue (w/w), supplemented with 2% (v/v) DTT and 2.5% iodoacetamide (w/w). The protein concentration was determined using the Bradford protein assay (B6916, Sigma, St. Louis, MO, USA). In order to quantify the SPA protein, different quantities of total soluble protein extracts (10, 20, 30, and 40 µg) were separated on SDS–polyacrylamide gel (T = 10.3%, C = 1.3%).

After electrophoresis, proteins were transferred onto a nitrocellulose membrane (Hybond, ECL, GE Healthcare) using a Criterion blotter (Biorad). The membrane was incubated for 1 h at room temperature in a blocking buffer containing 10 mM Tris–HCl pH 7.6, 150 mM NaCl, 0.01% (v/v) Tween 20, and 5% (w/w) skimmed milk. The membrane was then incubated for 1 h at room temperature and overnight at 4 °C with a 1:1000 dilution of anti-SPA antibody (Eurogentec S.A., Belgium). Antirabbit IgG conjugated with horseradish peroxidase (GE Healthcare) was used as the secondary antibody (diluted 1:50,000). The signal was detected using an Immobilon™ Western Chemiluminescent HRP Substrate (ECL Millipore) following the manufacturer’s protocol. Anti-SPA signals were quantified by image analysis using Image J software (http://imagej.nih.gov/ij). The fold-change in SPA protein abundance was calculated as the ratio of the slope of the relationship between protein amount and anti-SPA signal for the SPA RNAi and NS lines^45^.

### Data analysis

All statistical analyses were done in R-4.2.3 for Windows^46^ (code provided in the Supplementary Information). An ANOVA model with two factors (genotype and block) was used to analyze the results. Genotype and block were regarded as fixed effects. Variance homogeneity was tested using the Bartlett test and the normality of the residuals with the Shapiro–Wilk test. The block effect was never statistically significant. Differences between NS line and *SPA* RNAi line were tested using the post-hoc Dunnett test, with the NS line used as control. Statistical differences were judged at the 5% level. Differences in SPA protein abundance between NS line and *SPA* RNAi line were tested by comparing the slopes of the standard major axis regression between the normalized volume of anti-SPA signal and the total protein mass using the ‘smatr’ package^47^.

## Results

### *SPA* RNA and protein quantities are reduced in the *SPA* RNAi line

To investigate the role of SPA in regulating storage protein accumulation in bread wheat grain we generated RNAi transgenic lines to down regulate this gene. The transgene was under the control of the promoter of a HMW-GS gene. More precisely, the promoter of the allele encoding the Dx5 subunit of *Glu-D1-1* was used. This promoter is grain specific and highly induced during grain development^48^ (Fig. 1). We grew *SPA* RNAi plants and their null-segregant siblings (NS) in the greenhouse. Grain developmental stage was measured in degree-days (°C days) after anthesis to take into account the effect of temperature on development. At 300 °C days after anthesis the plants were either supplied with 15 mM nitrogen (N+ treatment) or no nitrogen (N− treatment) until the end of grain filling about 800 °C days after anthesis. We sampled grains every 100 °C days from 200 to 800 °C days after anthesis and then at maturity, 900 °C days and 1050 °C days after anthesis.

**Figure 2:**
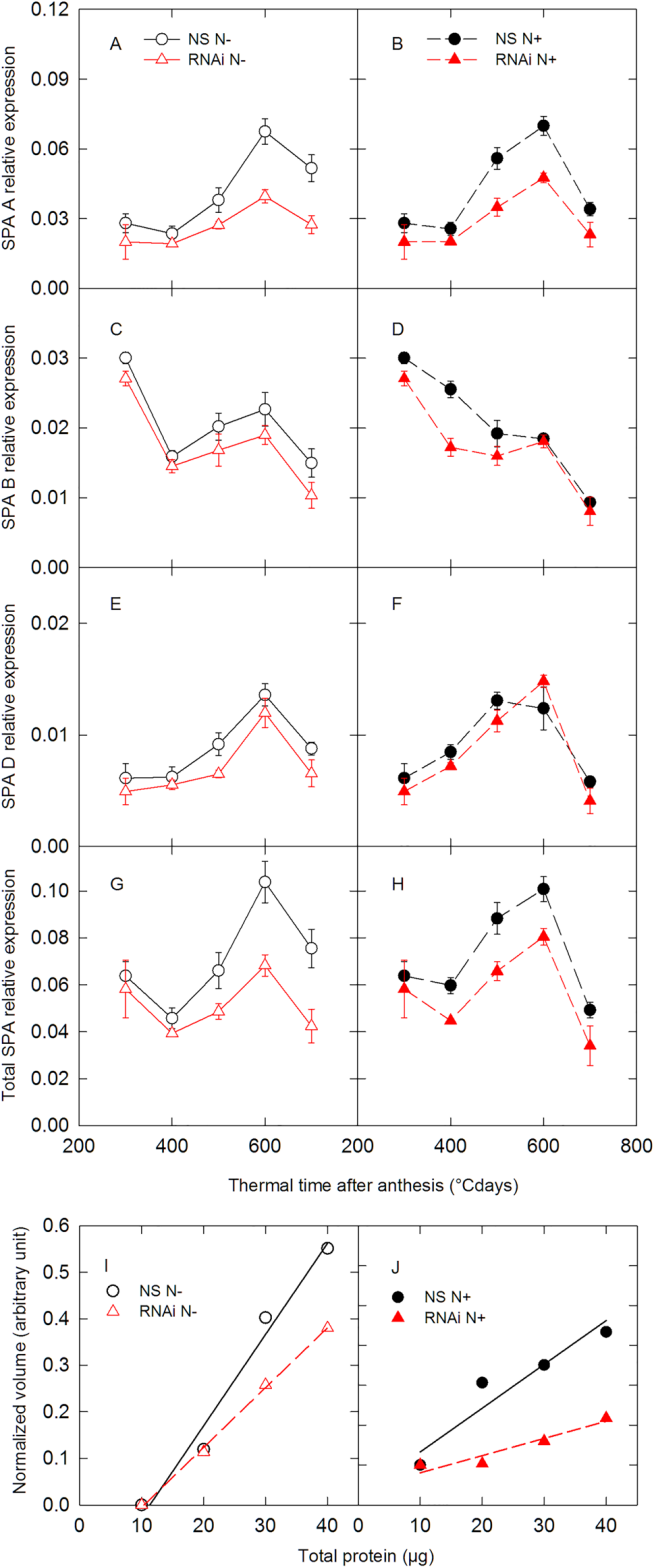
Down-regulation of *SPA* in the RNAi line. *SPA* null segregant (NS, circles) and RNAi (RNAi, triangles) lines of bread wheat were grown in the greenhouse with low (N−, open symbols) and high (N+ , closed symbols) nitrogen supply. (**A**–**F**) Relative expression of *SPA* homoeologs. (**G**) and (**H**) Relative expression of the sum of the three SPA homoeologs. (**I**) and (**J**) Quantification of the anti-SPA signal from western blots with different quantities of total protein extract at 500 °C days after anthesis. (**A**–**H**) Data are means for *n* = 4 independent replicates.

We demonstrated RNA interference in one of the transgenic lines generated by measuring the expression of the three *SPA* homoeologs during grain development using q-PCR (Fig. 2). In the RNAi line fewer *SPA-A* transcripts were detected throughout development under both nitrogen treatments compared to NS. As *SPA-A* is the most highly expressed of the three homoeologs, the decrease in its expression was largely responsible for the overall decrease in the relative abundance of *SPA* transcripts (Fig. 2G and H). For example, relative expression of total *SPA* transcripts was > 20% less in the RNAi line than in NS 500 °C days after anthesis for the N− treatment and 400 °C days after anthesis for the N+ treatment. Interestingly, *SPA-B* showed a different time-course of expression from *SPA-A* and *SPA-D* in both the NS and RNAi line. This difference has already been observed in different genetic backgrounds^26^ and suggests distinct regulation of the different homoeologs of *SPA*.

We also checked the effect of RNAi on SPA protein levels. We carried out a western blot analysis on grains collected 500 °C days after anthesis (Fig. 2I and J). The quantity of SPA protein was reduced by 34% (*P* = 0.071) for the N− treatment (Fig. 2I) and 61% (*P* = 0.057) for the N+ treatment (Figs. 2J and S3). Therefore less SPA protein accumulated when expression of *SPA* was down regulated by RNAi.

### Nitrogen accumulation is modified in the *SPA* RNAi line under low nitrogen availability

**Figure 3:**
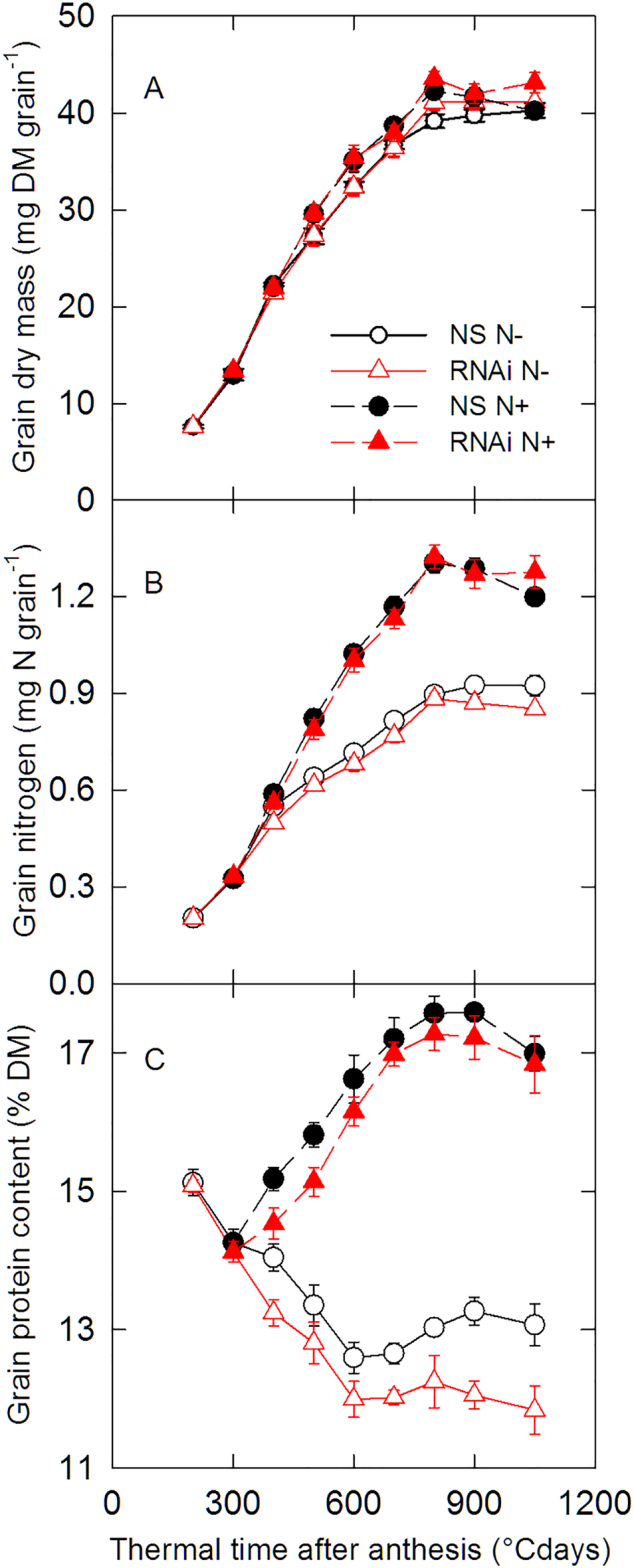
Changes in grain dry mass, total quantity of nitrogen (*N*_tot_) in the grain and grain protein concentration during grain development. *SPA* null segregant (NS, circles) and RNAi (RNAi, triangles) lines of bread wheat were grown in the greenhouse with low (N−, open symbols) and high (N+ , closed symbols) nitrogen supply. DM, dry mass. Data are means ± 1 s.e. for *n* = 4 independent replicates.

For plants subjected to the N− treatment, the total quantity of nitrogen per grain (*N*_tot_) and grain protein content (GPC) at maturity were reduced by 6% (*P* = 0.043) and 9% (*P* < 0.001) respectively in the *SPA* RNAi line compared with the NS. In the case of GPC, the decrease in the RNAi line compared to the NS was already significant (*P* < 0.05) at 400 and 700 °C days after anthesis (Fig. 3C). Under these conditions of low nitrogen availability, the greatest difference in GPC between *SPA* RNAi line and the NS was at maturity, which can be attributed to a late increase in single grain dry mass and a decrease in *N*_tot_ (Fig. 3A–C).

In contrast, for plants subjected to the N+ treatment, *N*_tot_ was not significantly different (*P* = 0.30) between the *SPA* RNAi and NS lines (Fig. 3B) and GPC was only significantly reduced (− 5%) in the RNAi line at 400 and 500°Cdays after anthesis (Fig. 3C). Grain yield per ear was increased by 9% while it was not significantly altered in the N− treatment.

Single grain dry mass at maturity was not significantly different between the *SPA* RNAi and NS lines for either of the nitrogen availability treatments (Table 1).

**Table 1:** Single grain dry mass, grain yield per ear, total quantity of N per grain (N_tot_), grain N yield per ear, and grain protein concentration at maturity (900 and 1050 °C days) for the null segregant (NS) and *SPA* RNAi (RNAi) lines under low (N−) and high (N+) nitrogen availability.

Variable	Treatment			
	N−		N+	
	NS	RNAi	NS	RNAi
Single grain dry mass (mg DM grain^−1^)	40.0 ± 0.5	41.2 ± 0.8	40.9 ± 0.6	42.6 ± 0.7
Grain yield per ear (g DM ear^−1^)	0.96 ± 0.03	1.05 ± 0.05	1.04 ± 0.02	1.14 ± 0.04*
Grain N (mg N grain^−1^)	0.92 ± 0.02	0.86 ± 0.01**	1.24 ± 0.02	1.27 ± 0.03
Grain N yield per ear (mg N ear^−1^)	22.4 ± 0.5	22.3 ± 0.8	32.0 ± 0.8	35 ± 1.3
Grain protein concentration (% of DM)	13.2 ± 0.2	11.9 ± 0.2***	17.3 ± 0.2	17.0 ± 0.3

Data are means ± 1 s.e. for *n* = 8 independent replicates. Within a single N treatment, * (*P* < 0.05), ** (*P* < 0.01) and *** (*P* < 0.001) indicate significant differences between values for NS and RNAi lines from an ANOVA followed by the Dunnett post-hoc test.

### Storage protein accumulation but not composition is affected in the *SPA* RNAi line under low nitrogen availability

**Figure 4:**
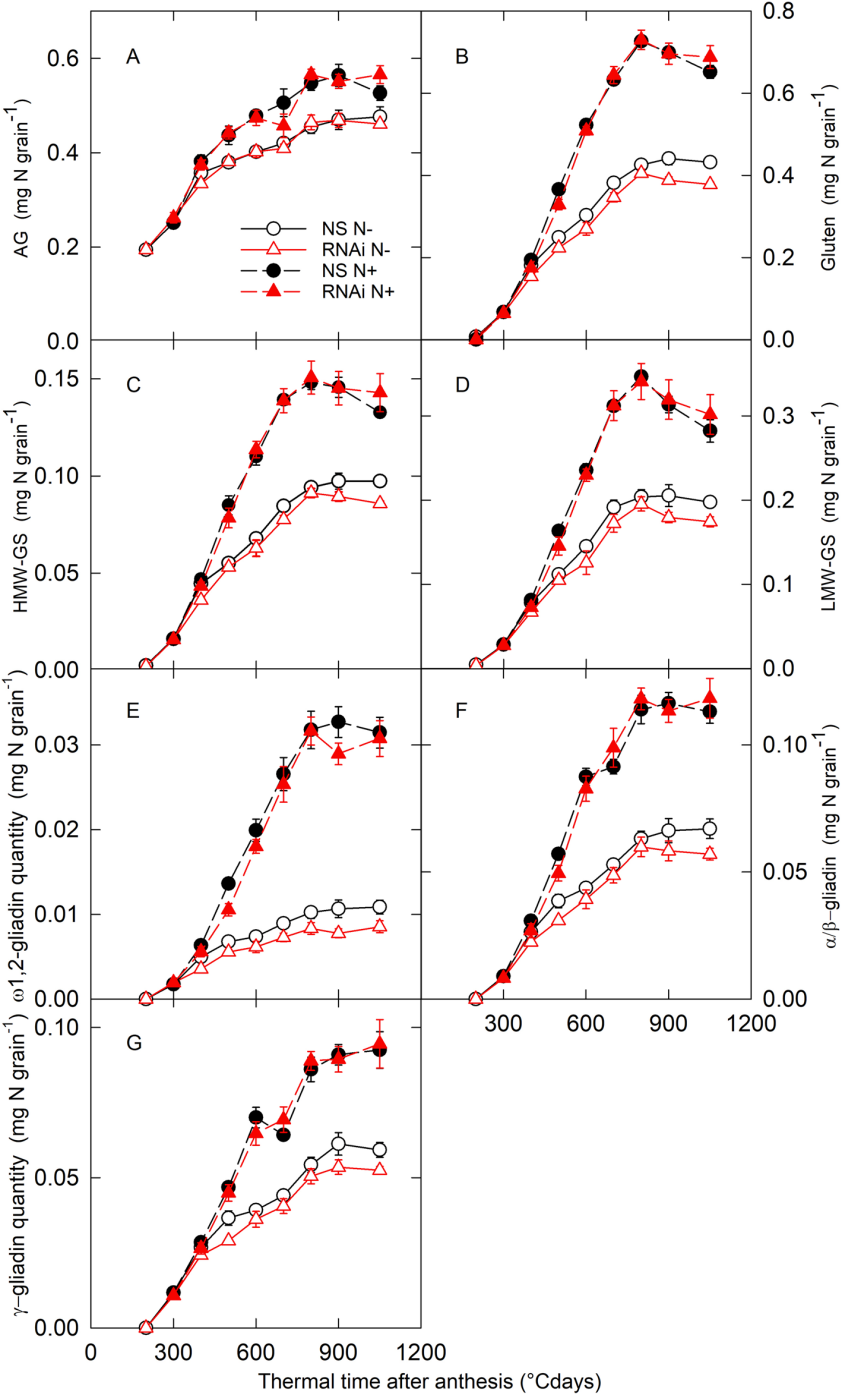
Changes in the quantity per grain of protein fractions during grain development. *SPA* null segregant (NS, circles) and RNAi (RNAi, triangles) lines of bread wheat were grown in the greenhouse with low (N−, open symbols) and high (N+, closed symbols) nitrogen supply. AG, albumin-globulin; HMW-GS, high molecular weight glutenin subunits; LMW-GS, low molecular weight glutenin subunits. Data are means ± 1 s.e. for *n* = 4 independent replicates.

We determined grain protein composition throughout grain development in the NS and *SPA* RNAi lines. Under N− treatment, decreases in all GSP fractions were measured from around 400 °C days and onwards in the *SPA* RNAi line compared to NS, while a slight decrease in the albumin-globulin (AG) fraction was only detected at maturity (Fig. 4). Decreases in GSPs must have contributed more to the overall decrease in GPC under low nitrogen availability as the GSP to AG ratio was lower in the *SPA* RNAi line than in NS (Table 2). In the N+ treatment, the glutenin subunits, gliadin classes and AG mostly accumulated at similar rates in the *SPA* RNAi and NS lines (Fig. 4) and at maturity the GSP to AG ratio was the same (Table 2). At maturity, for both N treatments, the gliadin to glutenin ratio tended to be lower (− 6%) in the *SPA* RNAi line than NS but the difference was not statistically significant (*P* = 0.5).

**Table 2:** Grain storage protein (GSP) to albumin-globulin (AG) ratio under low (N−) and high (N+) nitrogen availability and GSP composition at maturity (900 and 1050 °C days) under low nitrogen availability, shown as the percentage of high (HMW-GS) and low (LMW-GS) molecular weight glutenin subunit, ω1,2-gliadin, α/β-gliadin and γ-gliadin fractions in total GSP.

Variable	Treatment			
	N−		N+	
	NS	RNAi	NS	RNAi
GSP to AG ratio	1.59 ± 0.04	1.59 ± 0.04	1.59 ± 0.04	1.59 ± 0.04
HMW-GS (% GSP)	20.4 ± 0.24	21.0 ± 0.34	19.0 ± 0.52	18.5 ± 0.18
LMW-GS (% GSP)	42.4 ± 0.50	42.6 ± 0.52	40.6 ± 0.62	40.0 ± 0.28
ω1,2-gliadins (% GSP)	2.8 ± 0.08	2.4 ± 0.12**	5.3 ± 0.28	5.1 ± 0.10
α/β-gliadins (% GSP)	17.4 ± 0.31	17.0 ± 0.37	18.9 ± 0.54	19.7 ± 0.19
γ-gliadins (% GSP)	15.8 ± 0.28	15.7 ± 0.37	15.1 ± 0.35	15.6 ± 0.19

NS, null segregant line. RNAi, *SPA* RNAi line. Data are means ± 1 s.e. for *n* = 8 independent replicates. Within a single N treatment, ** (*P* < 0.01) indicate significant differences between NS and RNAi lines from an ANOVA followed by the Dunnett post-hoc test.

For N− conditions, the relative proportions of GSP fractions in total GSP were the same in the NS and *SPA* RNAi lines (Table 2), showing that all GSP fractions contributed to the same relative extent to the decrease in GSP quantity when *SPA* was downregulated. An exception was ω1,2-gliadin, which was reduced by 18% (*P* < 0.001) in the *SPA* RNAi line compared with NS. However ω1,2-gliadin makes up less than 4% of the total amount of GSP.

### *SPA* under-expression decreases gliadin gene expression more than glutenin gene expression

**Figure 5:**
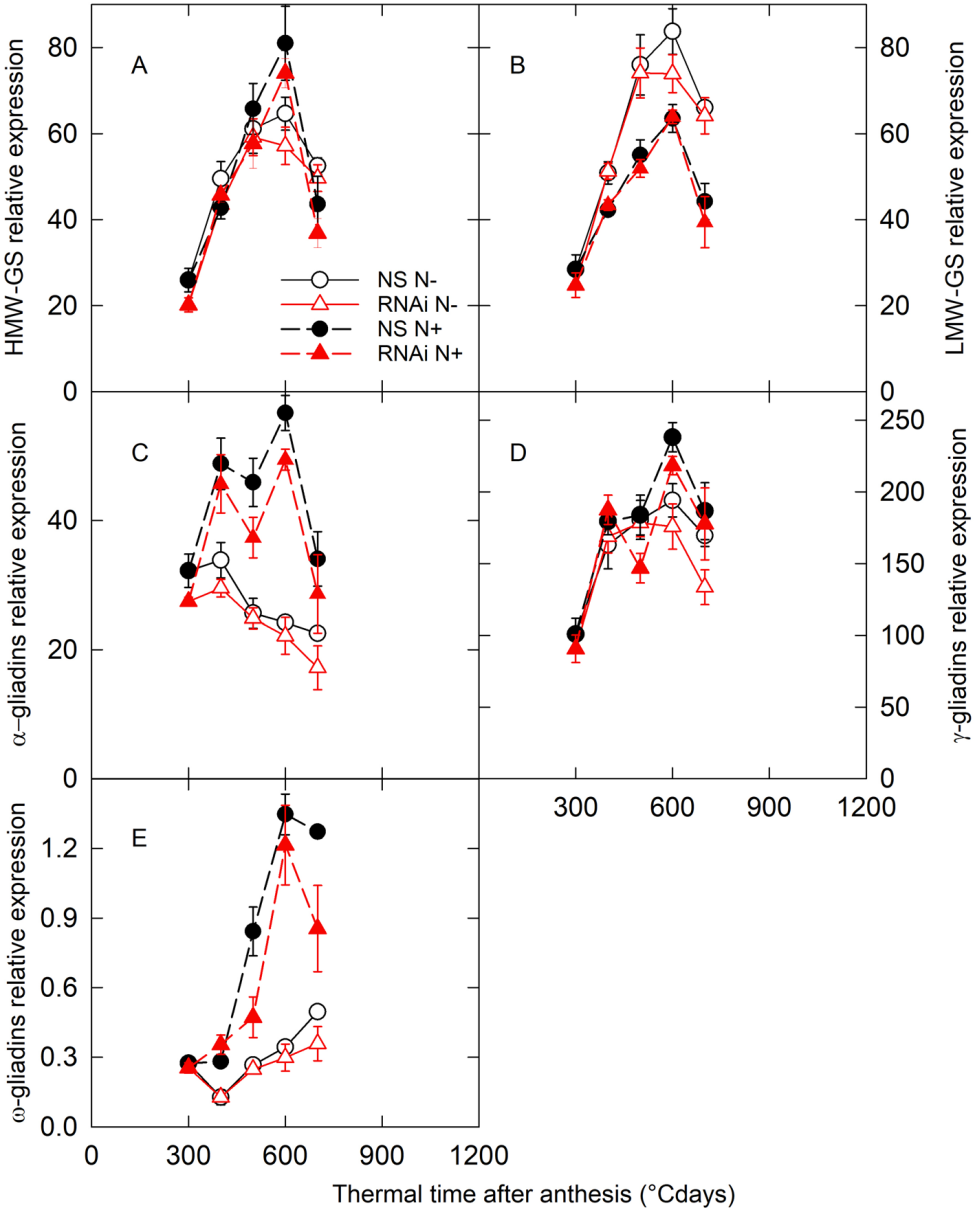
Changes in gene expression of grain storage proteins during grain development. *SPA* null segregant (NS, circles) and RNAi (RNAi, triangles) lines of bread wheat were grown in the greenhouse with low (N−, open symbols) and high (N+, closed symbols) nitrogen supply. HMW-GS, high molecular weight glutenin subunits; LMW-GS, low molecular weight glutenin subunits. Data are means ± 1 s.e. for *n* = 4 independent replicates.

As in bread wheat SPA is a transcriptional regulator of GSP genes^13^, we measured the expression of genes belonging to the different GSP families in the wheat *SPA* RNAi line (Fig. 5). For most families we used generic q-PCR primers to amplify transcripts of all the genes of a given family. For HMW-GS we amplified transcripts of the four HMW-GS genes expressed in the line used for transformation separately then summed the result. Our results show that all gene families were down regulated in the *SPA* RNAi line compared to NS for at least one time point of either of the treatments. In N+ conditions, the expression of some GSP genes was affected in the *SPA* RNAi line, mainly at the later time points (500 °C days after anthesis and onwards). The time points coincide with the largest differences in *SPA* expression in the RNAi line (Fig. 2). In the N+ treatment, glutenin genes were the least affected in the *SPA* RNAi line with no change in expression detected for LMW-GS, while gliadin genes showed the most striking decrease in expression (Fig. 5). Similar results were observed for the N− treatment but the differences between the *SPA* RNAi line and the NS line were smaller than for the N+ treatment.

**Figure 6:**
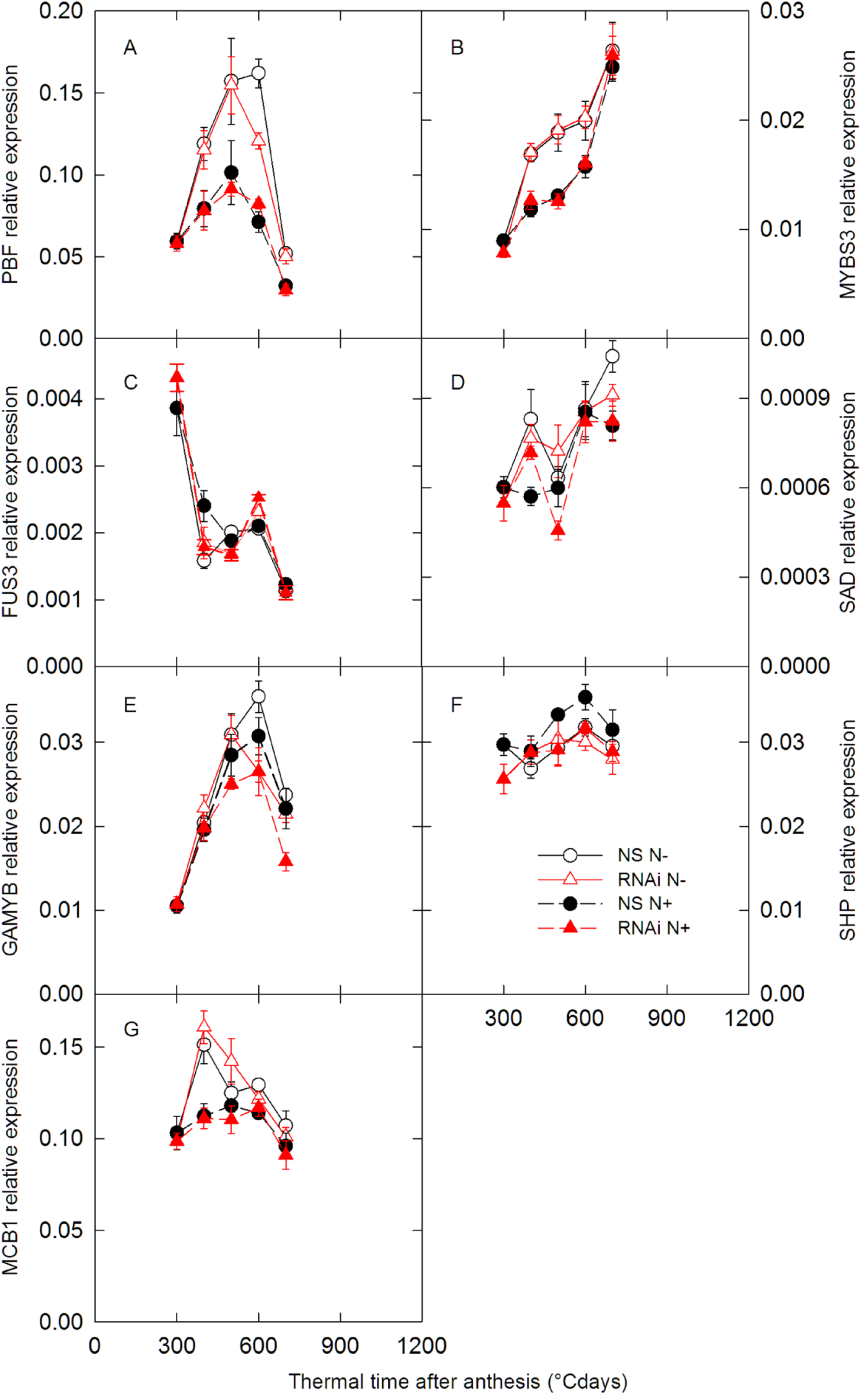
Changes in gene expression of transcription factors of the grain storage protein transcriptional regulation network during grain development. *SPA* null segregant (NS, circles) and RNAi (RNAi, triangles) lines of bread wheat were grown in the greenhouse with low (N−, open symbols) and high (N+, closed symbols) nitrogen supply. Full details of gene names are provided in the text. Data are means ± 1 s.e. for *n* = 4 independent replicates.

Expression of TFs known to be part of cereal GSP regulatory network was also quantified during the linear grain filling period (Fig. 6). The expression of *PBF*, *MYBS3*, *GAMYB* and *MCB1* was lower in N+ than in N− conditions in both *SPA* RNAi and NS lines. The expression of *SAD* and *SHP* was upregulated in the N+ treatment compared with the N− treatment in NS for at least two time points, but in the *SPA* RNAi line *SHP* did not respond to nitrogen supply. *PBF* and *GAMYB* expression was downregulated for one or more time points in the *SPA* RNAi line compared with NS under N− conditions, while *SHP* and *GAMYB* were downregulated in the *SPA* RNAi line compared with NS under N+ conditions for at least one time point.

## Discussion

In this study, we show that the under-expression of *SPA* in bread wheat can result in reduced expression of GSP genes and when nitrogen is limiting a decrease in GPC and the GSP to AG ratio of grain. These results confirm previous indications that SPA has a role in the transcriptional regulation of GSP genes^13^ like its orthologs in other cereals^11,28,30^. However, down-regulating *SPA* had a fairly limited effect on GSP gene expression. This may have been because the decrease in *SPA* gene expression was not large enough to induce a stronger effect or because of functional redundancy like in rice where the under-expression of both *RISBZ1*, the *SPA* ortholog, and *RPBF* resulted in a much more significant decrease in GSP gene expression than in the *RISBZ1* knock-down line^29^.

Our results show that *SPA* under-expression has a stronger effect on the expression of gliadin than glutenin genes. This is consistent with a study of natural genetic variation in wheat where two haplotypes of *SPA-*A were identified. Different levels of *SPA-*A expression from each haplotype led to the allocation of different quantities of total grain nitrogen to the gliadin fraction, but equal amounts to the glutenin fraction^26^.

SPA activates the expression of LMW-GS and HMW-GS by binding the GLM^9,10,49^, so we expected to observe a lower level of glutenin expression in the *SPA* RNAi line. However, HMW-GS and LMW-GS gene expression was barely affected by *SPA* under-expression (Fig. 5A and B). For both HMW-GS and LMW-GS, the level of gene expression we measured here was the combined expression of several members of the gene family. Nucleotide diversity in promoters of LMW-GS gene family members^50^ could however result in differences in expression patterns. As we were not able to monitor the expression of each individual LMW-GS gene, particularly the one used in previous studies^9,49^, the known effect of SPA regulation might have been masked by different responses from the rest of the gene family. The GLM motifs identified in the promoters of HMW-GS genes were shown to be functional as they are activated after SPA binding^10^; the same applies to the G-like box with bZIP proteins^48^. As these boxes belong to a common regulatory framework shared by all the HMW-GS promoters^10^, it is expected that the entirety of HMW-GS genes respond in the same way.

Changes in GSP gene expression alone did not account for all of the modifications in GSP accumulation observed in the *SPA* RNAi line under low nitrogen supply. In addition differences in gene expression did not always lead to differences in protein accumulation. For example, gene expression of gliadins was lower in the *SPA* RNAi line than in the WT under high nitrogen availability at some time points, but this did not result in any detectable change in grain protein accumulation or composition. This indicates that mechanisms other than transcription regulate GSP synthesis compensating for the deregulation of GSP expression. Discrepancies between gene expression and protein accumulation in wheat have already been observed for γ-gliadins in a study of the effect of nitrogen and sulfur availability on the regulation of wheat GSP synthesis^34^ and for gliadins as part of the characterization of *nac019* triple mutants^25^. Moreover, an association study of wheat grain protein composition found that several nitrogen assimilation and metabolism genes were associated with GPC and *N*_tot_, further evidence of levels of non-transcriptional regulation of grain protein accumulation^41^. Field studies have shown that *N*_tot_ is mainly determined by the supply of N to grains and its accumulation is therefore mainly source driven^35,51,52^.

The effect of under-expressing *SPA* was dependent on nitrogen availability as we only observed changes in grain protein content and composition under the low nitrogen treatment. This does not seem to be related to *SPA* being more highly expressed in the RNAi line when nitrogen was available, as on the contrary, there was still an obvious decrease in SPA protein concentration compared to the control. GLM binding by SPA has already been shown to have a role in integrating the effects of nitrogen availability at the transcriptional level on GSP synthesis in barley grain^38^. Here we saw in wheat that this occurs mostly at the protein synthesis level. In *o2*, a maize mutant for the *SPA* ortholog *O2*, genes involved in amino acid metabolism are differentially expressed^28^. Under varying levels of nitrogen and sulfur availability, amino acid transport and metabolism are modulated to adjust wheat GSP synthesis and composition^34^. Thus SPA probably modulates the nitrogen response at different levels, both directly by regulating GSP gene expression and indirectly by controlling the expression of other genes involved in regulating GSPs either transcriptionally, as suggested by our results, or translationally^53^.

We attempted to generate plants over-expressing *SPA* but none of the transformed lines showed the expected increase in *SPA* gene expression. This could mean that over-expression of *SPA* is detrimental to the development of the embryo or to germination. Our use of an HMW-GS gene promoter for the *SPA* transgene may have resulted in a lethal dose of SPA as it contains an activation domain for *SPA* itself that might have generated a feed-forward regulatory loop. Another study was more successful in producing SPA over-expressors in bread wheat, achieving ten to 20-fold increases in the expression of *TaSPA-B*^31^; while they used an HMW-GS promoter like us, it came from a different allele and it is unclear which part of the promoter they used, therefore it is possible the absence of some boxes led to lower, and thus non-lethal, levels of *SPA* expression than in our transformants. Unexpectedly in regards to our results and previous studies^13^, Guo et al.^31^ found that the over-expression of *SPA* did not lead to higher accumulation of GSP, and on the contrary diminished the quantity of glutenin and ω-gliadin. This could be at least partly due to the indirect effect of SPA over-expression reducing the expression of the glutenin activator *PBF*^16^ and increasing the expression of the glutenin repressor *SHP*^13^. The study by Guo et al.^31^ was done at a single level of nitrogen supply and our results, along with previous work^38^, show that SPA regulation of GSP accumulation and the expression of other transcription factors in the regulatory network is dependent on nitrogen availability. It is thus possible that different consequences of increasing the expression of *SPA* would have been obtained varying nitrogen supply and that using plants with modified expression of SPA might improve GPC under certain conditions of fertilization. More generally, any attempt at improving GPC in cereals should involve testing in a wide range of nutritional conditions.

### Supplementary Information

Supplementary Information.

## Data Availability

The datasets generated during the current study are available from the corresponding author on reasonable request.
