# Supplementary material for: Storage protein activator controls grain protein accumulation in bread wheat in a nitrogen dependent manner
Source: Sci Rep. 2023 Dec 20;13:22736. doi: 10.1038/s41598-023-49139-5 (PMC10733432; doi:10.1038/s41598-023-49139-5)
Supplement: Supplementary file 1 — Supplementary Information. [file 41598_2023_49139_MOESM1_ESM.docx]

**Supplementary figures**


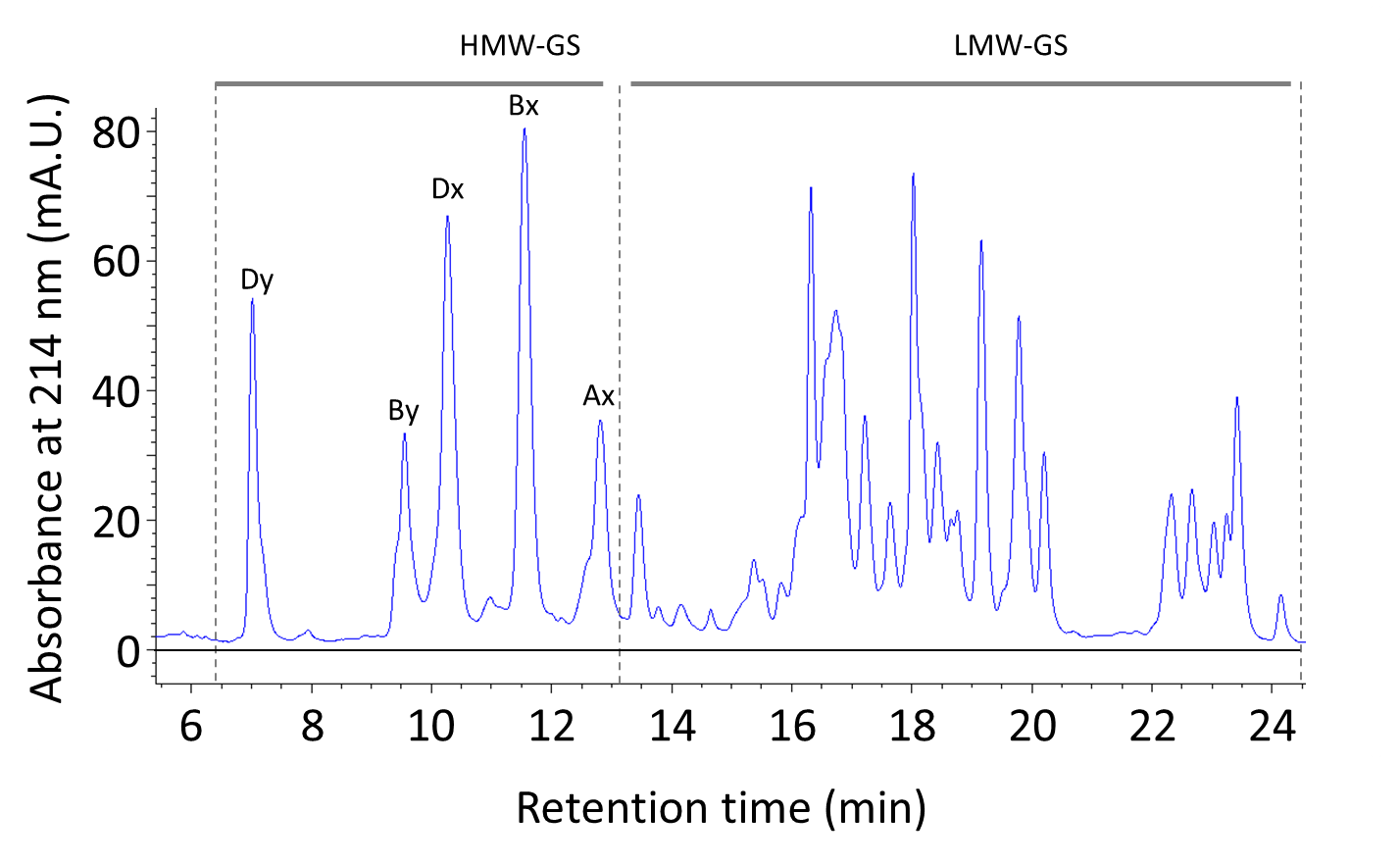


**Figure S1.** HPLC chromatogram of glutenin from whole meal flour of the *Triticum aestivum* line NB1. The peaks corresponding to high molecular weight (HMW-GS) and low molecular weight (LMW-GS) glutenin subunits are indicated.


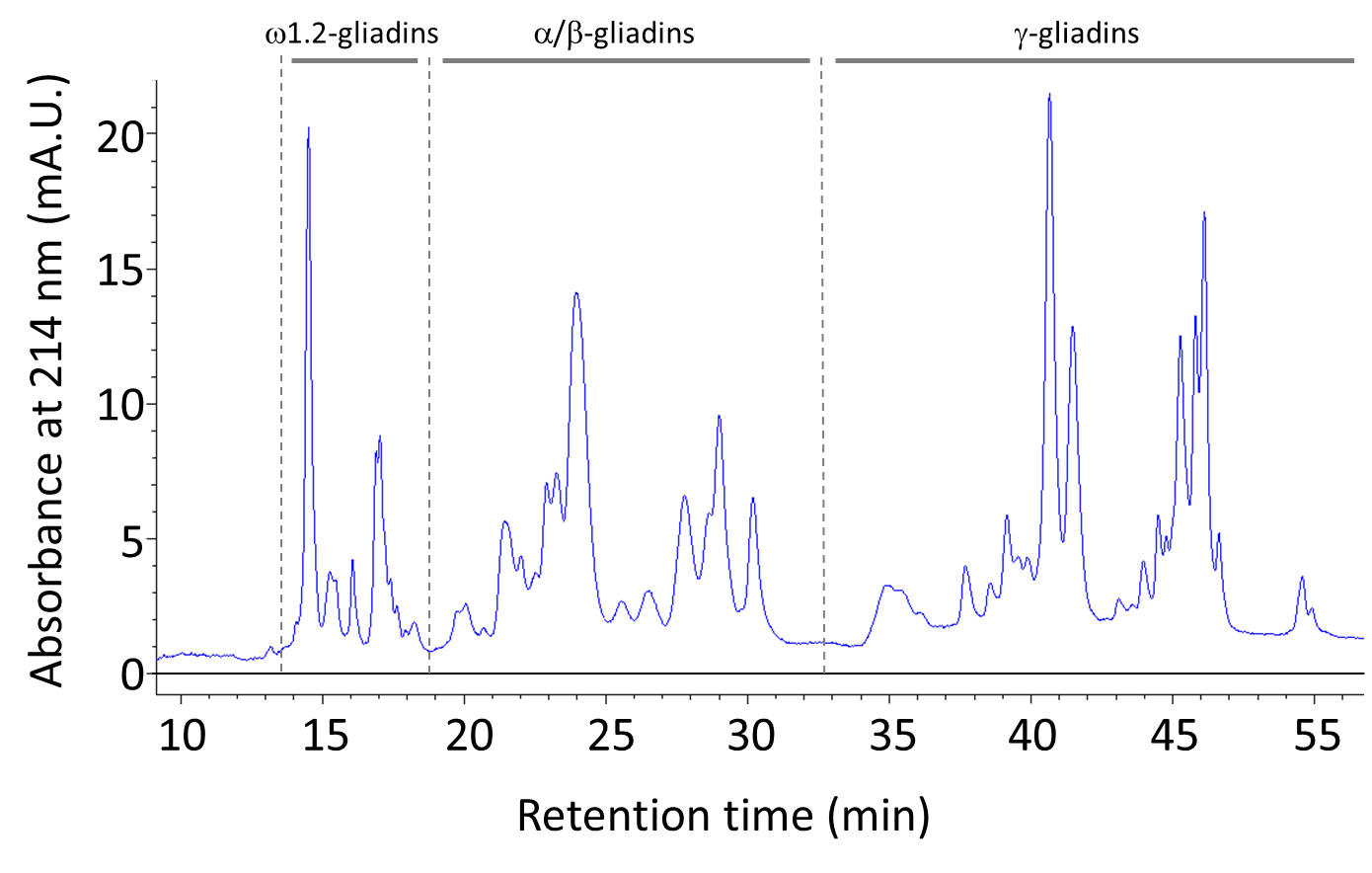


**Figure S2.** HPLC chromatogram of gliadin from whole meal flour of the *Triticum aestivum* line NB1. The peaks corresponding to ω1,2-, α/β-, and γ-gliadin are indicated.


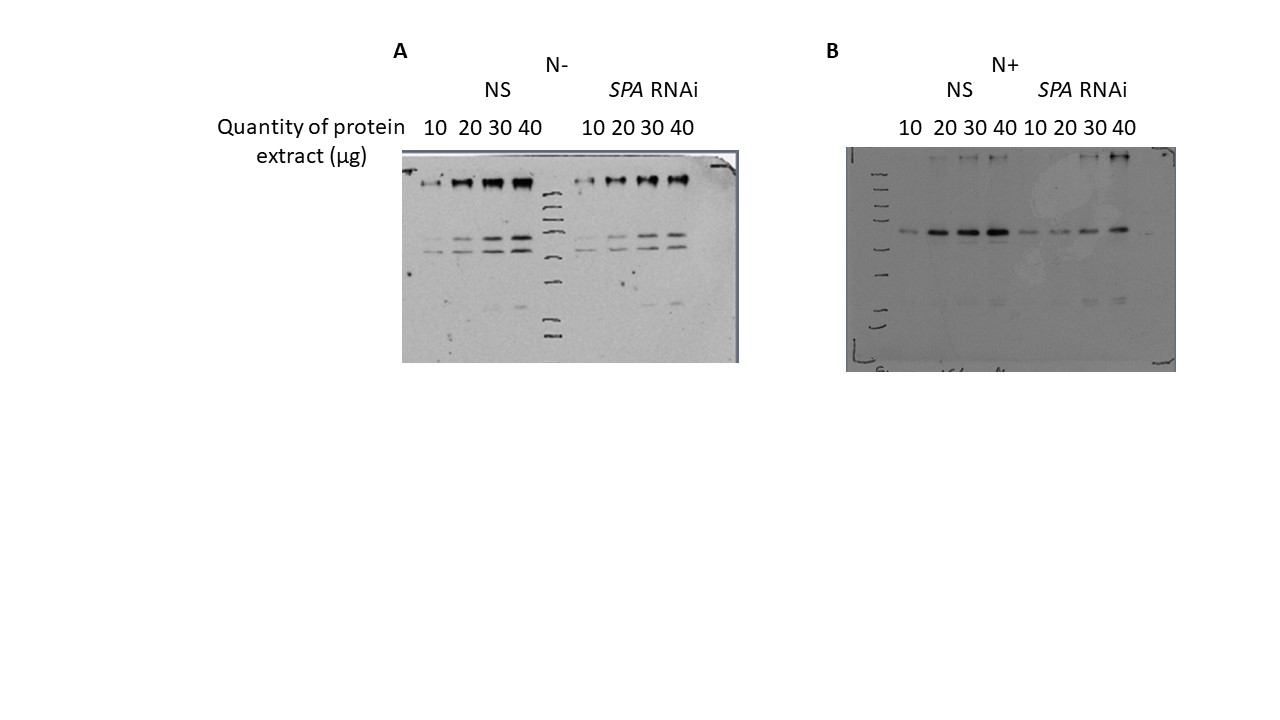


**Figure S3**. Quantification of the anti-SPA signal from western blots with different quantities of total protein extract at 500°Cdays after anthesis. *SPA* null segregant (NS) and RNAi lines of bread wheat were grown in the greenhouse with A. low (N-) and B. high (N+) nitrogen supply.

**Supplementary tables**

| **Table S1** Segregation in the T1 generation for *SPA* RNAi. This was established by cultivating 30 plants on a medium with kanamycin to identify homozygotes (resistant), hemizygotes and null segregants | | | | |
| --- | --- | --- | --- | --- |
| Percentage of homozygotes | | Percentage of null segregants | |  |
| 15% | | 23% | |  |
| **Table S2** Sequences of primers used for q-PCR. The first three genes are housekeeping genes used for normalization of relative gene expression. | | | | |
| Gene | Forward primer (5’–3’) | | Reverse primer (5’–3’) | |
| *β-tubulin* | CCATCAGTTGGTTGAGAATGC | | CAAAGCTGGGAGTGGTCA | |
| *GAPDH* | TTCAACATCATTCCAAGCAGC | | CGTAACCCAAAATGCCCTTG | |
| *eF1α* | CAGATTGGCAACGGCTACG | | CGGACAGCAAAACGACCAAG | |
| *SPA*-A | GATCATCACCCGCAAATGA | | AACAAGCCGAGAAACCACTATA | |
| *SPA*-B | CCAAGCAATGTCTAGCATATCATC | | GGTTGCATATGTAGCGAAGGT | |
| *SPA*-D | TCCGCAACGGGAATCTACTA | | AATGTCCACTTAACAAGCCAAACT | |
| *HMW-GS Ax* | CATGCCGACAGGTCGTAG | | CTGTTGCGGAGAAGTTACACTTA | |
| *HMW-GS Bx* | GGTGCCGCCCCATCAC | | GCAGGTATTCCCCAAAATATCAT | |
| *HMW-GS Dx* | AGCGGTTAGTCCTCTTTGTGG | | CGGAGCTGCTGGTCCATG | |
| *HMW-GS Dy* | GTTAGCGCAGAGCAGCAAG | | CCCTCCATCCGACACACTG | |
| *LMW-GS* | CAGCTAAACCCATGCAAGGTATTC | | TGCGACCTAGCAAGACGTTGT | |
| *ω-gliadin* | AACAACCATTCCCCAGACC | | AATGATTGTTGTGGTTGCACG | |
| *α-gliadin* | ATTGCAGCCACAAAATCC | | TGGTCGAAATGGTTGTGG | |
| *γ-gliadin* | AAATCCACCATGAAGACCTTACT | | TGTGGTTGCTGGGAGAATGG | |
| *PBF* | AGAAGAAGCCTCGGCCAAAG | | CAGAACTTGGTGTTGCCAGACTT | |
| *SHP* | GACAATTTCCTCAACGAGGC | | AGGTGCTCCAGGCTCGC | |
| *FUSCA3* | CAAGTGGCTGGGTTGCGAG | | CCATCCTTTGATGTCAGAATTG | |
| *MCB1* | GAACTTCGTCACCACCAGG | | GAGTATCTTCTCAGCAGGAGCA | |
| *SAD* | GCAACTCCACCAACACCAAG | | GAGCGAGCCGCCCTC | |
| *GAMYB* | CTCAGGCCCAACCTCAAGAAG | | TTGGAGTGGAGCTGGATGATG | |
| *MYBS3* | CGTGCAGGGATCATCATC | | TGTTCTTCTTCAGTCCAA | |

**Supplementary information**

Code used for the statistical analysis.

library('readxl')

library(multcomp) # For Dunnett multi-comparison test

library(doBy) # to calculate mean and s.e.

library(tidyverse)

############infile#####################################

mat.Data <- read_excel("DataMat.xlsx", sheet="Pheno_comp")[1:32,c("bloc","genotype","ST","treatment", "p.N", "p.C", "GPC", "GNC", "EDW", "PS.lyoph.an","GDW.an", "HM.SP", "LM.SP", "w1_2gli.SP", "a.gli.SP", "g.gli.SP", "SP_AG")]

mat.DataN=mat.Data[mat.Data$treatment=="N+",]

mat.DataN0=mat.Data[mat.Data$treatment=="N-",]

##########################designing outfiles#########################################

res.stat <- data.frame(matrix(data = NA, nrow = 11, ncol = 2))

rownames(res.stat) <- c( "GPC", "GNC", "EDW", "PS.lyoph.an","GDW.an", "HM.SP","LM.SP","w1_2gli.SP","a.gli.SP","g.gli.SP","SP_AG")

colnames(res.stat) <- c("N-","N+")

trait=names(mat.Data[,7:17])

pvalueN<-data.frame(t(rep(NA, 7)))

colnames(pvalueN)<-c("trait","SCEtot", "%SCEgeno","%SCEbloc","%SCE res","pgeno","pbloc") #####N+

pvalueN0<-data.frame(t(rep(NA, 7)))

colnames(pvalueN0)<-c("trait","SCEtot", "%SCEgeno","%SCEbloc","%SCE res","pgeno","pbloc") #####N0

Norm <- data.frame(matrix(data = NA, nrow = 11, ncol = 2)) #shapiro test on residues

rownames(Norm) <- c( "GPC", "GNC", "EDW", "PS.lyoph.an","GDW.an", "HM.SP","LM.SP","w1_2gli.SP","a.gli.SP","g.gli.SP","SP_AG")

colnames(Norm) <- c("N-","N+")

VarHom<-as.data.frame(matrix(data = NA, nrow = 11, ncol = 2)) #variance homogeneity

rownames(VarHom) <- c( "GPC", "GNC", "EDW", "PS.lyoph.an","GDW.an", "HM.SP","LM.SP","w1_2gli.SP","a.gli.SP","g.gli.SP","SP_AG")

colnames(VarHom) <- c("N-","N+")

#########################Analysis#################################################

#factors definition

mat.DataN$bloc <- as.factor(mat.DataN$bloc)

mat.DataN$genotype <- as.factor(mat.DataN$genotype)

mat.DataN0$bloc <- as.factor(mat.DataN0$bloc)

mat.DataN0$genotype <- as.factor(mat.DataN0$genotype)

#interaction

mat.Data$geno.treat=interaction(mat.Data$genotype, mat.Data$treatment)

#anova

index <- 0

for (t in 1:length(trait))

{

ant<-aov(formula(paste(trait[t]," ~ (genotype )",sep="")),data=mat.DataN)

ant2<-aov(formula(paste(trait[t]," ~ (genotype + bloc )",sep="")),data=mat.DataN)

ti= summary(ant2)

ta=unlist(ti[[1]])

SCEtot=sum(ta[4:6])

pvalueN[t,]=c(trait[t], SCEtot,

round(ta[4]/SCEtot,3),round(ta[5]/SCEtot,3),round(ta[6]/SCEtot,3),

round(ta[13],5),round(ta[14],5))

titi=glht(ant,linfct = mcp(genotype = "Dunnett"))

tata=confint(titi, level = 0.95)

dunnett = summary(tata)

# dunnett <- summary(confint(glht(ant) ,

# linfct = mcp(genotype = "Dunnett")) , level = 0.95)

index <- index + 1

# P-value from dunnett test

res.stat[t,"N+"] <- round(dunnett$test$pvalues[[1]] ,4)

#residue normality

shapN<-shapiro.test(ant2$residuals)

Norm[t,"N+"]<-as.numeric(shapN[2],4)

x <- rnorm(1000,0,1)#1000 observations de moyenne 0 et de variance 1 (loi normale)

hist(rstandard(ant2),freq = FALSE,main = trait[t])

plot(function(x) dnorm(x,0,1), xlim=c(-3,3),add=TRUE)

#variance homogeneity

b = unlist(bartlett.test(formula (paste(trait[t],"~ genotype"), sep=""), data=mat.DataN))

VarHom[t,"N+"]=as.numeric(b["p.value"])

}

rm(ti,ta, SCEtot, titi, tata,b)

#for N-

pvalueN0<-data.frame(t(rep(NA, 7)))

colnames(pvalueN0)<-c("trait","SCEtot", "%SCEgeno","%SCEbloc","%SCE res","pgeno","pbloc")

index <- 0

for (t in 1:length(trait))

{ ant<-aov(formula(paste(trait[t]," ~ (genotype )",sep="")),data=mat.DataN0)

ant2<-aov(formula(paste(trait[t]," ~ (genotype + bloc )",sep="")),data=mat.DataN0)

ti= summary(ant2)

ta=unlist(ti[[1]])

SCEtot=sum(ta[4:6])

pvalueN0[t,]=c(trait[t], SCEtot,

round(ta[4]/SCEtot,3),round(ta[5]/SCEtot,3),round(ta[6]/SCEtot,3),

round(ta[13],5),round(ta[14],5))

titi=glht(ant,linfct = mcp(genotype = "Dunnett"))

tata=confint(titi, level = 0.95)

dunnett = summary(tata)

#dunnett <- summary(confint(glht(ant) ,

# linfct = mcp(genotype = "Dunnett")) , level = 0.95)

index <- index + 1

# P-value from dunnett test

res.stat[t,"N-"] <- round(dunnett$test$pvalue[1] ,4)

shapN0<-shapiro.test(ant2$residuals) #residue normality

shapN<-shapiro.test(ant2$residuals)

Norm[t,"N-"]<-as.numeric(shapN[2],4)

x <- rnorm(1000,0,1)

hist(rstandard(ant2),freq = FALSE,main = trait[t])

plot(function(x) dnorm(x,0,1), xlim=c(-3,3),add=TRUE)

b = unlist(bartlett.test(formula (paste(trait[t],"~ genotype"), sep=""), data=mat.DataN0)) #variance homogeneicity

VarHom[t,"N-"]=as.numeric(b["p.value"])

}

##mean and standard deviation calculations

Moy= summaryBy(.~geno.treat , keep.names = T , FUN = mean , data = mat.Data , na.rm = T) #mean

Et= summaryBy(.~geno.treat , keep.names = T , FUN = sd , data = mat.Data , na.rm = T) #standard deviation
